# Segmental Colitis Associated With Diverticulosis Masquerading as Polyploid-Appearing Mucosa in the Rectosigmoid Area on Endoscopy and as Focal Thickening on Imaging

**DOI:** 10.7759/cureus.22930

**Published:** 2022-03-07

**Authors:** Eugene C Nwankwo, Gebran Khneizer, Gregory Sayuk, Jill Elwing, Necat Havlioglu, Michael Presti

**Affiliations:** 1 Internal Medicine, Saint Louis University School of Medicine, Saint Louis, USA; 2 Gastroenterology and Hepatology, Saint Louis University School of Medicine, Saint Louis, USA; 3 Gastroenterology and Hepatology, Saint Louis Veterans Affairs Medical Center, Saint Louis, USA; 4 Division of Gastroenterology and Hepatology, Washington University School of Medicine, Saint Louis, USA; 5 Department of Pathology, St. Louis Veteran’s Administration, St. Louis, USA; 6 Division of Gastroenterology and Hepatology, Saint Louis University School of Medicine, Saint Louis, USA

**Keywords:** diverticulosis, colitis, inflammatory bowel disease, inflammatory disorders, luminal disease

## Abstract

Segmental colitis associated with diverticulosis (SCAD) is an inflammatory disease affecting segments of the large bowel with diverticular disease. SCAD presents several challenges in diagnoses and treatment because it often mimics a range of disorders including inflammatory bowel disease and malignancy. Here, we present the case of a 72-year-old man with lower abdominal pain and bloody stools whose initial abdominal workup showed nonspecific large bowel thickening and concerns for malignancy. Ultimately, the patient was diagnosed with mild SCAD and treated conservatively with a resolution of symptoms. He had no symptoms at the three-month and 1-year follow-ups. This case highlights the importance of including SCAD in the initial differential diagnosis to allow accurate identification and treatment.

## Introduction

Segmental colitis associated with diverticulosis (SCAD) is an inflammatory disease affecting segments of the large bowel with diverticular disease [1-3]. SCAD typically involves the sigmoid colon and can present with rectal bleeding, tenesmus, or changes in bowel habits. Further, it can mimic a malignancy or inflammatory bowel disease (IBD) [1,3,4]. The clinical progression of the disease is benign and often managed conservatively with antibiotics and/or 5-aminosalicylic acid therapy and rarely leads to complications. In this case report, we present the case of an elderly patient who reported left lower abdominal pain, fevers, and chills. He was found to have evidence of left colonic diverticulitis on imaging, followed by endoscopic evaluation showing malignant-appearing polyploid mucosa in the rectosigmoid region.

## Case presentation

**Figure 1:**
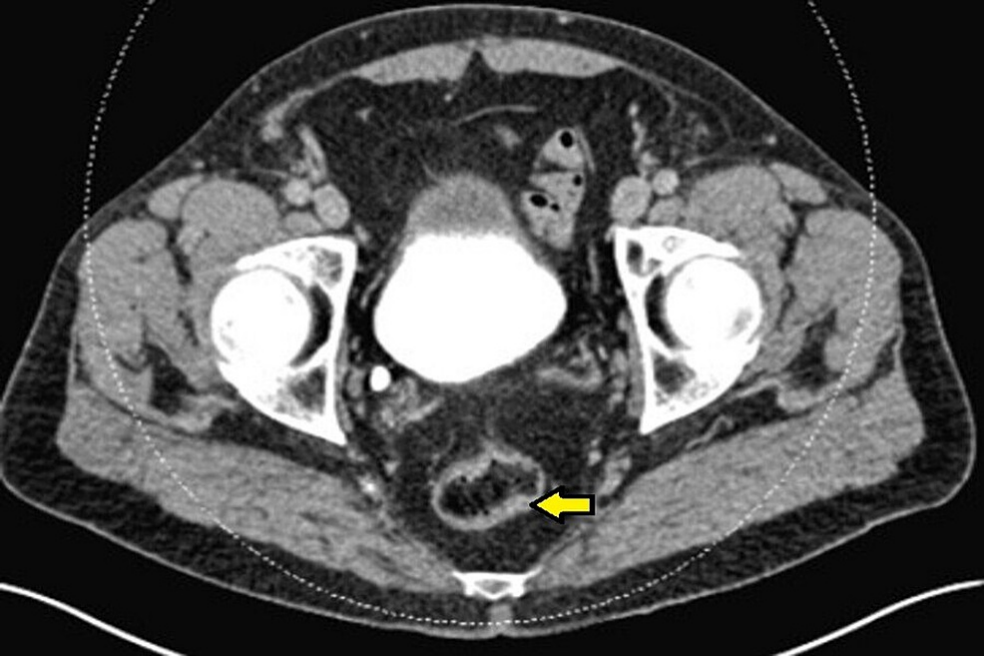
CT of the abdomen and pelvis revealed abnormal thickening of the sigmoid colon (yellow arrow). Initial workup for lower abdominal pain and intermittent hematochezia revealed a thickened rectosigmoid with a broad differential diagnosis. CT: computed tomography

**Figure 2:**
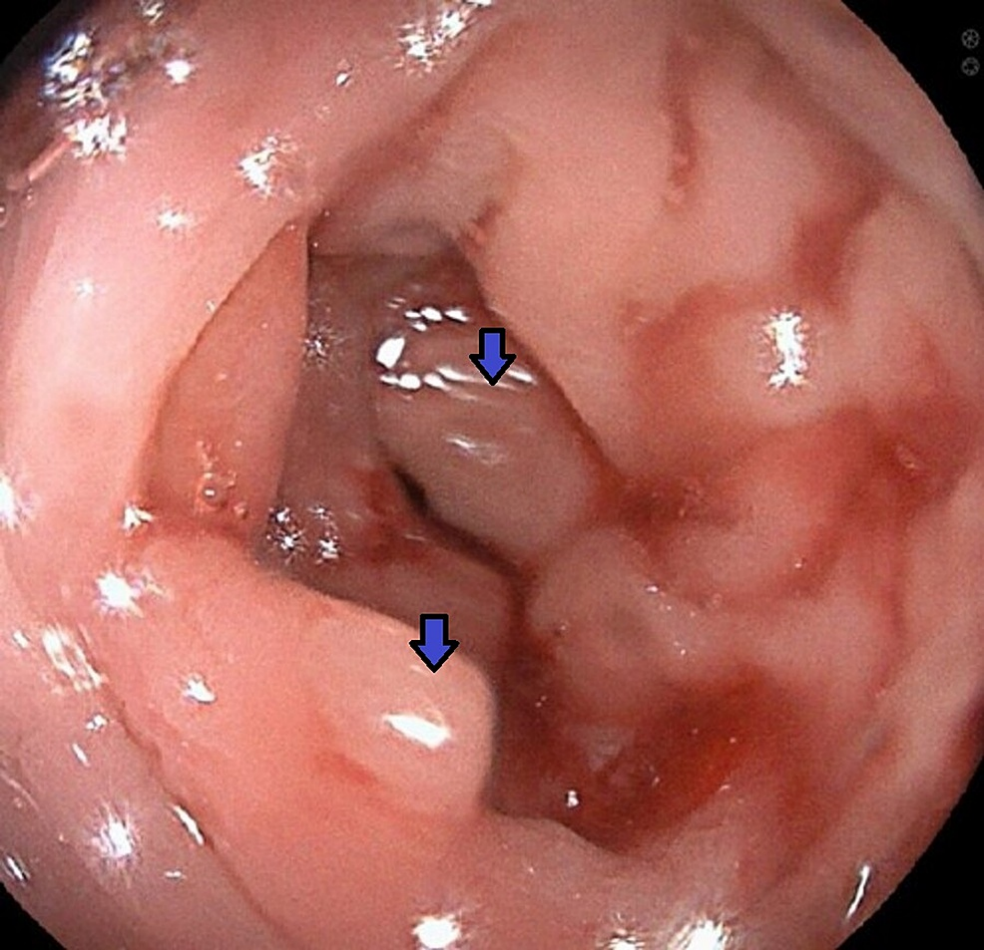
Polyploid-appearing mucosal region (blue arrow) in the rectosigmoid area on initial colonoscopy. Initial CT findings of the thickened sigmoid colon were correlated with endoscopy and biopsy that showed evidence of polyploid-appearing mucosa in the rectosigmoid region. The pathology indicated moderate active colitis with cryptitis and crypt abscess with inflammatory changes consistent with resolving diverticulitis and no evidence of malignancy. CT: computed tomography

**Figure 3:**
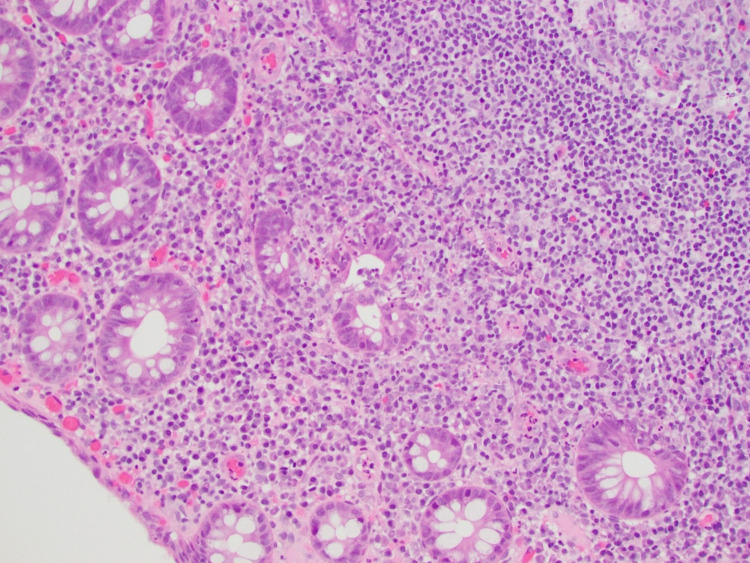
High-power micrograph of colonic mucosa which exhibits moderate active colitis, with cryptitis and crypt abscesses. Benign-appearing lymphoid aggregates are noted. There is no evidence of malignancy.

**Figure 4:**
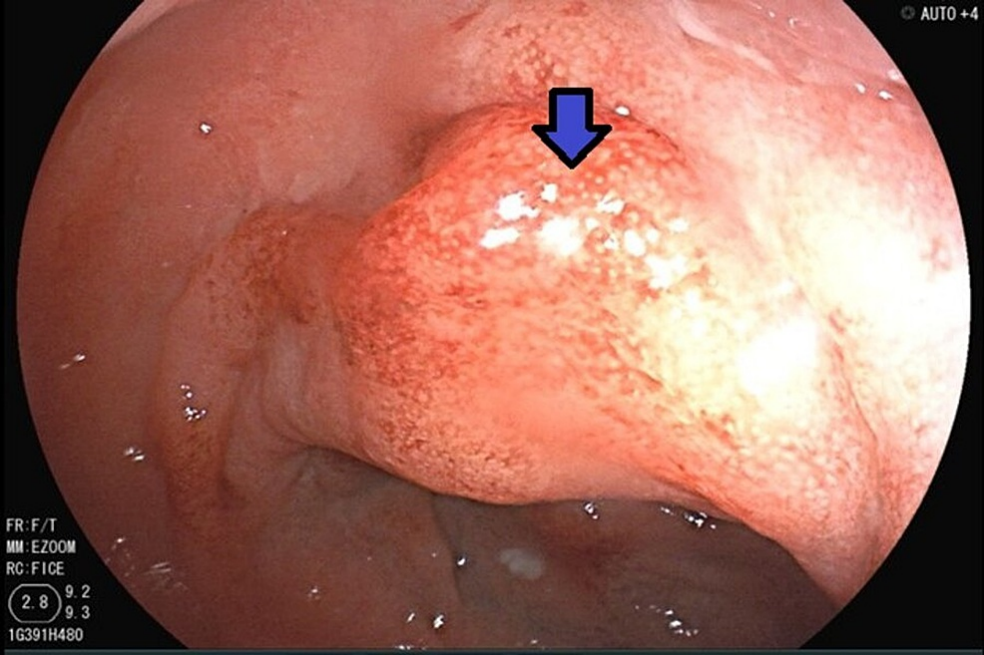
Area of colitis characterized by swollen erythematous mucosa without ulceration associated (blue arrow) with moderate diverticulosis on follow-up flexible sigmoidoscopy. Three months after discharge, endoscopic evaluation of the sigmoid revealed persistent stigmata of inflammation without gastrointestinal symptoms.

**Figure 5:**
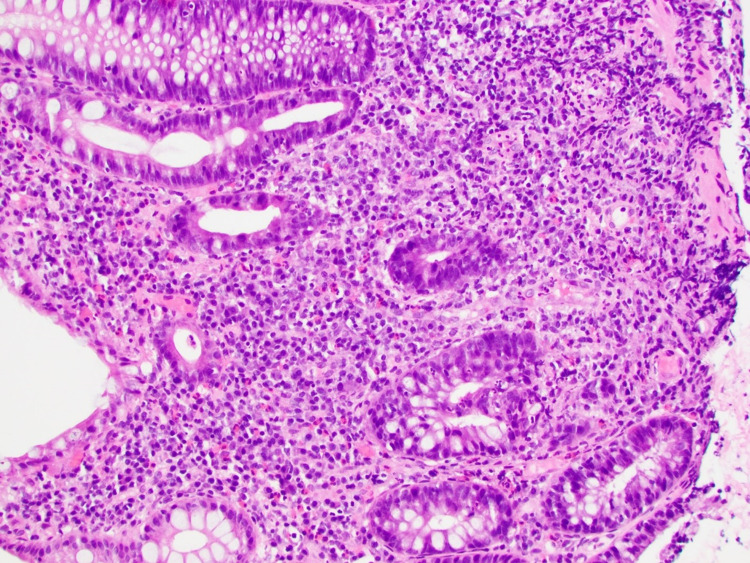
High-power micrograph of the colon biopsy shows crypt architectural distortion, diffuse marked active chronic inflammation, and foci of acute cryptitis and crypt abscesses. Granuloma formations and dysplasia are not seen.

A 72-year-old man with a medical history of glaucoma, hyperlipidemia, and diabetes mellitus type 2 was evaluated for a one-day history of left lower abdominal pain and intermittent bloody stools. His complaints were associated with progressive abdominal pressure and discomfort for the same duration. The patient’s vitals were remarkable for a blood pressure of 150/84 mmHg, normal heart rate, and temperature. Laboratory testing including complete blood count and biochemistry were unremarkable. Computed tomography (CT) of the abdomen and pelvis revealed an abnormal thickening of the sigmoid colon concerning for acute sigmoid diverticulitis or malignancy (Figure 1). One month later, a colonoscopy identified a polyploid-appearing mucosal region in the rectosigmoid area corresponding to the abnormal area of imaging, and biopsies were obtained (Figure 2). The initial suspicion was for a colon malignancy. The pathology returned moderate active colitis with cryptitis and crypt abscess with inflammatory changes consistent with resolving diverticulitis. There was no evidence of malignancy (Figure 3). Follow-up flexible sigmoidoscopy three months later revealed an area of colitis characterized by swollen erythematous mucosa without ulceration associated with moderate diverticulosis (Figure 4). Biopsies at the time of the flexible sigmoidoscopy showed findings consistent with those from the initial colonoscopy (Figure 5). Given his mild severity of symptoms, the patient was not treated and monitored with follow-up imaging. Repeat CT of the abdomen and pelvis eight months later revealed persistent pericolic hyperemia and minor nodal prominence in the proximal sigmoid colon. The patient was clinically asymptomatic on follow-up at three-month and one-year intervals.

## Discussion

SCAD is an inflammatory disease affecting segments of the large bowel with a diverticular disease that typically involves the sigmoid colon and can mimic a malignancy or IBD. It can present with a wide range of symptoms such as rectal bleeding, diarrhea, and abdominal pain. Segmental colitis (or diverticular colitis) is commonly associated with varying complications such as bleeding, perforation, and formation of abscesses [5]. The term segmental colitis is used to indicate the co-occurrence of chronic mucosal inflammation and diverticulosis [6]. The prevalence of segmental colitis is estimated between 0.3% and 1.4% of all patients with diverticulosis. The clinical presentation of SCAD is nonspecific and consists of rectal bleeding, left-sided abdominal pain, and less frequent changes in stool texture.

Visualization of the involved mucosa further allows for the subclassification of SCAD into one of four types (type A to D) by histologic appearance. Histology ranges from crescentic fold pattern (type A) to severe ulcerative colitis-like pattern (type D) [7-9]. However, SCAD can rarely present in atypical forms including pseudo polyps or post-inflammatory polyps. Our patient’s evaluation revealed a polyploid-appearing mucosal region in the rectosigmoid area which was initially thought to be adenomatous in nature with a concern for a malignant process. The concern for adenoma was initially high due to the patient’s age and prior diagnosis of IBD.

This case highlights the need to include SCAD as part of the differential diagnosis. Treatment of SCAD is not clearly defined. Therapy is geared toward reducing mucosal inflammation in moderate-to-severe SCAD with mesalamine, corticosteroids, and antibiotics [9]. Physicians should maintain a high index of suspicion for SCAD as a possible explanation for abnormal colonic thickening.

## Conclusions

SCAD can present with a broad spectrum of symptoms. Although visualization of the involved mucosa is the best means for diagnosing the disease, care must be taken to avoid misclassification based on endoscopic or radiographic findings including colonic thickening. Therefore, physicians must maintain a high suspicion for SCAD as a possible explanation for abnormal colonic thickening.
